# The Role of Inflammatory Mediators in Colorectal Cancer Hepatic Metastasis

**DOI:** 10.3390/cells11152313

**Published:** 2022-07-27

**Authors:** Lavanya Goodla, Xiang Xue

**Affiliations:** Department of Biochemistry and Molecular Biology, University of New Mexico School of Medicine, Albuquerque, NM 87131, USA; lgoodla@salud.unm.edu

**Keywords:** chemokines, colorectal cancer, cytokines, liver metastasis

## Abstract

Colorectal cancer (CRC) is the second leading cause of death in cancer patients in the USA, whereas the major cause of CRC deaths is hepatic metastases. The liver is the most common site of metastasis in patients with CRC due to hepatic portal veins receiving blood from the digestive tract. Understanding the cellular and molecular mechanisms of hepatic metastases is of dire need for the development of potent targeted therapeutics. Immuno-signaling molecules including cytokines and chemokines play a pivotal role in hepatic metastases from CRC. This brief review discusses the involvement of three representative cytokines (TNF-α, IL-6 and IL-1β), a lipid molecule PGE2 and two chemokines (CXCL1 and CXCL2) in the process of CRC liver metastases.

## 1. Introduction

Colorectal cancer (CRC) constitutes 10.2% of tumor-related morbidity and 9.2% of tumor-related mortality worldwide [1]. Fifty percent of CRC patients are prone to foster the state of hepatic metastases during their disease, and these metastases are the major cause of deaths [2,3]. Metastasis is a process of collective steps including shedding from the primary tumor, invasion into and survival in the circulation, combat against host defense systems, arrival at a new site, extravasation into the tissue and proliferation at the new site [4]. However, the mechanisms regulating CRC liver metastasis is not fully understand. Portal drainage of the gastrointestinal tract partially contributes to the excessive rate of hepatic metastasis in CRC. At the same time, ancillary molecular variables are certainly crucial in ascertaining whether CRC can metastasize into the hepatic tissue.

Tumor cells are obsessive to the inflammatory stroma, indicating that the tumor microenvironment (TME) is the lucrative target for a therapeutic approach. The stromal cells in the TME can secrete factors such as chemokines to recruit inflammatory cells, which can produce soluble cytokines to aid cancer cell survival by avoiding the effect of host defense mechanisms [5,6,7]. Elevated levels of cytokines such as tumor necrosis factor alpha (TNF-α), interleukin (IL)-6 and IL-1β, a lipid molecule prostaglandin E2 (PGE2), and chemokines such as CXC chemokine ligand 1 (CXCL1) and CXCL2 in the serum of CRC patients were associated with cancer development and progression [8,9,10]. Thus, cytokines, lipid molecules and chemokines are potential targets for anticancer therapies. The current review evaluates the mechanistic roles of cytokines and chemokines in the process of CRC hepatic metastasis.

## 2. Cytokines

Cytokines are signaling molecules that play a pivotal role in divulging within the immune system and in permitting the exchange of information between immune system and host tissue cells. Cytokines bind to receptors and trigger signal cascades in the recipient cells to modify the functions or phenotypes. These complex signal cascades are able to interconnect a wide range of environmental factors. A variety of cytokine families exist which are structurally related but perform divergent functions. Targeting the cytokines is a robust proven method in various ailments, and yet many of the cytokines are under investigation as therapeutic agents or targets [11,12,13]. 

## 3. TNF-α

### 3.1. TNF-α Discovery

As its name suggests, TNF-α was originally identified in 1975 as an anticancer agent that causes endotoxin-induced hemorrhagic necrosis in sarcoma and other tumors, and human TNF-α was cloned in 1984 [14,15]. It has been postulated that TNF-α executes a pivotal function in inflammation and cancer [16]. TNF-α is produced as the transmembrane inactive protein of 26-kilodaltons (kDa) in the plasma membrane of the cell processed by a TNF-α-converting enzyme which aids in cleaving the extracellular domain of the TNF-α precursor to release the soluble active protein of 17 kDa TNF-α [17]. Secreted TNF-α functions in an autocrine/paracrine manner [18]. 

### 3.2. TNF-α Expression and Regulation in Primary Colon Tumor and Hepatic Metastasis

The major source of production of TNF-α in CRC is activated macrophages [18,19]. Natural killer cells, T lymphocytes, dendritic cells (DCs) and epithelial cells can also produce TNF-α [20]. The increased expression of TNF-α in CRC tissues and serum are positively associated with tumor recurrence, advancement and metastasis leading to decreased survival of CRC patients [21,22]. The expression of TNF-α can be regulated by hypoxia at the transcriptional level. Specifically, we demonstrated that TNF-α is a direct target gene of hypoxia inducible factor (HIF)-2α in intestinal epithelial cells [20]. Sullivan et al. demonstrated that TNF-α is regulated by epigenetic modifications such as DNA methylation, relocation of the TNF-locus into euchromatin, Histone H3 lysine 9 (H3K9) methylation, histone acetylation and Histone H3 lysine 4 (H3K4) methylation at the TNF-α locus [23]. 

### 3.3. TNF-α Function and Therapeutic Targeting Potential

**Figure 1:**
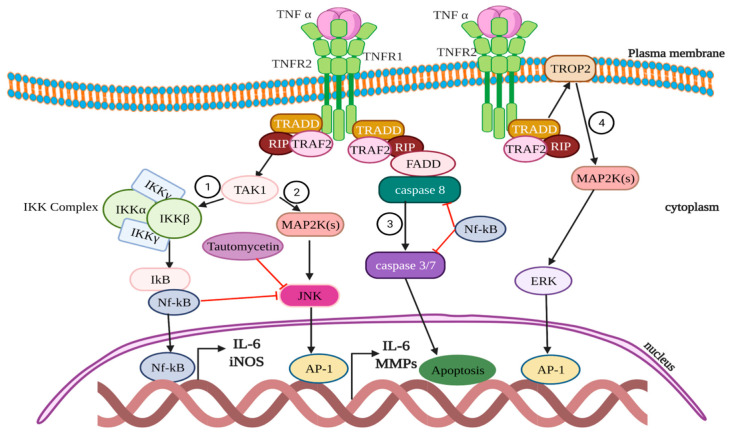
Schematic representation of TNF-α-signaling pathway in CRC. (**1**) NF-KB: The Binding of TNF-α to TNFR1 facilities the assembly of TNFR1-associated death domain (TRADD) complex containing TRAF2 and RIP, which leads to activation of TAK1 and I kappa B kinase complex (IKK), comprised of two kinases IKKα and IKKβ, and eventually increases nuclear accumulation of NF-kB. (**2**) AP-1: After binding to TNFR1, TNF-α triggers the phosphorylation of mitogen-activated protein 2 kinases (MAP2Ks) and in turn activates Jun-N-terminal kinase (JNK) and activator protein-1 (AP-1). (**3**) Cell death: TNF-α causes apoptosis or necrosis through caspase enactment by binding to TRADD complex to FADD. (**4**) ERK: Enhancement or upregulation of TROP-2 by TNF-α through activating ERK pathway.

The signaling of TNF-α is through the TNF receptor (TNFR). TNFR1 is expressed in most cells, whereas TNFR2 is limited to certain cell types (myeloid cells, regulatory T-cells and glial cells) and some endothelial cell types. Moreover, TNFR2 can be upregulated in epithelial cells, fibroblasts and certain T- and B-cell subsets [24]. The enactment of the TNF-α-signaling cascade downstream of the TNFR1 occurs through following four major mechanisms [25] (Figure 1): (1) NF-KB: The Binding of TNF-α to TNFR1 facilities the assembly of TNFR1-associated death domain (TRADD) complex containing TRAF2 and receptor-interacting protein (RIP), which leads to activation of transforming growth factor beta-activated kinase 1 (TAK1) and I kappa B kinase complex (IKK), comprised of two kinases IKKα and IKKβ, and eventually increases nuclear accumulation of nuclear factor kappa-light-chain-enhancer of activated B cells (NF-kB), thereby leading to cell survival [26]. (2) AP-1: After binding to TNFR1, TNF-α can also trigger the phosphorylation of mitogen-activated protein 2 kinases (MAP2Ks) and in turn activates Jun-N-terminal kinase (JNK) and activator protein-1 (AP-1) to promote CRC [27,28]. (3) Cell death: TNF-α causes apoptosis or necrosis through caspase enactment by binding to TRADD complex to the FAS-associated death domain (FADD). (4) Extracellular signal-regulated kinase (ERK): Another pathway or mechanism of action for CRC cell migration and invasion was significant enhancement or upregulation of the tumor-associated calcium signal transduction protein (TROP-2) by TNF-α through activating the extracellular signal-regulated kinase (ERK) pathway [29]. It was also proposed that TNF-α-induced TNFR2 expression triggers the proliferation of CRC cells and intestinal epithelial cells by stimulating the signal transducer and activator of transcription-3 (STAT3) protein for tumor promotion [24,30]. TNF-α can also promote accumulation and survival of the myeloid derived suppressor cells (MDSCs) via TNFR2 signaling [31]. 

A few studies demonstrate the dual role of TNF-α in colorectal carcinoma. On one hand, TNF-α promotes cancer cells survival by activating TNFRs, vascular cell adhesion molecule 1 (VCAM1) and altering the protein complex I and II (complex I consists of TRADD, TRAF2 and RIP and Complex II that consists of TRADD, RIP, FADD and caspase-8) as described above [32,33]. On the other hand, TNF-α can also act as a tumor suppressor via rebuilding the TME by enhancing cytotoxic T cell activity, promoting DCs maturation, and preventing tumor angiogenesis. Mice deficient in both TNF-α and IL-10 spontaneously develop severe colitis-associated colon cancer [34,35]. Increased TNF-α concentration in tumor-infiltrating lymphocytes and CRC tissue is an independent factor of better survival [34,35]. Macrophage TNFR2 is critical to the antitumor effect of TNF, potentially via increasing nitric-oxide-mediated inhibition effects of tumor angiogenesis [36]. The clear contribution of TNF-α to CRC tumor development and progression can be categorized by studying its secretion time and the type of immune cells secreting it during the process of tumor formation. It was reported that hematopoietic cell-derived TNF-α executes a key function in the production of intestinal polyps in adenomatous polyposis coli *(Apc)^Δ468^* mice, which is a sporadic CRC model [37]. Similarly, a strong decrease in tumor progression was exhibited when TNF-α was blocked in mice induced with colon tumors by azoxymethane (AOM) and dextran sulfate sodium (DSS) [38]. 

A reduction in the development of tumors can be achieved by TNF-α antibody neutralization blockade, knockout of TNFR1, or NF-kB inhibition [39,40]. Treatment with TNF-α antagonists Enbrel or Remicade ameliorates HIF-2α promoted colitis, which is a high-risk factor for CRC [20]. Furthermore, a recent population-based cohort study in Denmark on more than 56,000 inflammatory bowel disease patients reported that there is no significant risk of cancer when treated with TNF-α antagonists such as infliximab, adalimumab, and certolizumab pegol [41]. This study indicates that TNF-α antagonists may reduce the risk of colitis-associated CRC. 

Mice with intrasplenic or portal injection of metastatic human CRC CX-1 cells showed an increase in TNF-α and IL-1α production by activated Kupffer cells, which resulted in an increase in the sinusoidal endothelial cell adhesion molecules and trans-endothelial migration of the tumor cells. Blocking TNF-α signaling reduces the number of hepatic metastases induced by human CRC CX-1 cells. Similarly, another report states that TNF-α stimulates IL6 and granulocyte colony-stimulating factor production, which boosts the Kupffer cell activation and promotes neutrophil infiltration in hepatocytes, thereby enhancing the CRC liver metastasis [42]. In a clinical study, Infliximab treatment is safe and well-tolerated in patients with advanced cancer, including 12 CRC, 8 ovary cancer and 4 renal cancer patients [43]. 

## 4. Interleukin-6 (IL-6)

### 4.1. IL-6 Discovery

At first, IL-6 was studied by different research groups with different nomenclatures, such as B-cell stimulatory factor 2 (BSF-2), hepatocyte-stimulating factor, hybridoma growth factor and interferon (IFN)-β2, based on its biological activity [44,45,46]. After the successful cloning of BSF-2 cDNA in 1986, it was identified by various groups that all studied a single molecule with different names, and hence coined a single name: IL-6. Structurally, IL-6 is a four-α-helix protein with the molecular weight ranging from 21–26 kDa. Human IL-6 is a protein of 212 amino acids, and its gene is mapped to chromosome 7p21 [47]. 

### 4.2. IL-6 Expression and Regulation in Primary Colon Tumor and Hepatic Metastasis Tissue

IL-6 is generated by diverse cell types (macrophages, monocytes stromal, hematopoietic, epithelial and muscle cells) [48]. In TME, it is secreted by a wide range of cell types such as fibroblast stromal cells, tumor infiltrating immune cells and the tumor cells themselves [49]. It is well-established that tumor-associated macrophages (TAMs) enhance the cancer progression and metastasis via the release of a various cytokines including IL-6. In addition to preventing primary T-cell activation, MDSCs secret IL-6 to attenuate functional differentiation of the tumor-specific cluster of differentiation 4^+^ (CD4^+^) T cells into effector Th1 cells and promote tumor progression [50]. Preoperative serum IL-6 higher than 10 pg/mL is a predictor of poor prognosis for survival, independent from tumor site, grade and stage in CRC patients [51]. CRC patients with decreased IL-6 expression in the primary stage of the tumors show prolonged disease-free survival [52], whereas increased IL-6 expression is associated with the advanced stage of CRC and decreased survival rate in the patients [53].

CRC liver metastasis is supported by cancer-associated fibroblasts which are recruited from pericryptal and distant fibroblast precursors to produce a prometastatic microenvironment through inflammatory activation of IL-6 and the monocyte chemo-attractant protein-1 (MCP-1) [54,55,56,57]. Inflammatory fibroblasts from human CRC liver metastasis produce an increased amount of IL-6 and MCP-1 in comparison with nontumor liver tissue fibroblasts, which aids in liver metastasis [58]. IL6 is induced by various factors such as TNF-α, NF-κB and hypoxia [59,60,61]. TNF-α in cancer-related fibroblasts and hepatic fibroblasts strongly triggers the IL-6 and MCP-1 expression levels, which in turn create a prometastatic microenvironment [58]. In another report, it was represented that TNF-α, by binding to the TNFRs, triggers the production of NF-κB, which triggers the production of IL-6 [26]. Xu et al. reported that in CRC, hypoxia induces IL-6 expression, and thereby triggers the IL-6/STAT3/Bcl2 pathway and treatment resistance. They have also stated that the HIF-1a/miR-338-5p/IL-6 feedback loop, which was the result of STAT3/Bcl2 activation, was essential for drug resistance in CRC cells [59]. 

### 4.3. IL-6 Function and Therapeutically Targeting Potential 

**Figure 2:**
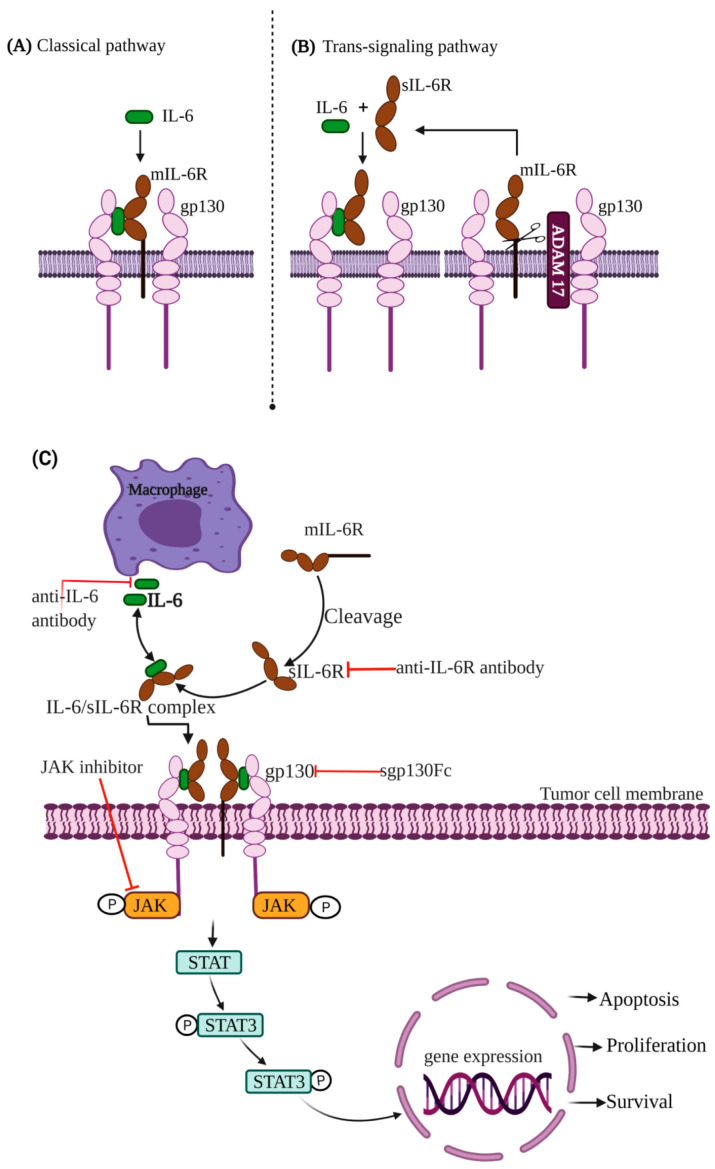
Schematic representation of IL-6-signaling pathways in CRC. (**A**) Classical signaling pathway, IL-6, binds to the membrane-bound IL-6R (mIL-6R), which then transmits the signal by recruiting and homodimerizing two glycoprotein 130 (gp130) subunits. (**B**) Cells which are not able to secrete mIL-6R, react to IL-6 through the alternative pathway, which is referred as the IL-6 trans-signaling pathway. In this pathway, a soluble receptor of IL-6 (sIL-6R) is released by alternative splicing or by A Disintegrin and Metalloprotease 17 (ADAM17) protease-mediated receptor shedding from the membrane. (**C**) The binding of the IL-6/mIL-6R or IL-6/sIL-6R complex to gp130 on tumor cells activates Janus kinases (JAKs) and STAT3 signaling and promotes tumor cell proliferation.

IL-6 has pleiotropic effects including immune response, inflammation and hematopoiesis. It is well-documented that IL-6 is one among the key cytokines involved in inflammatory bowel disease and CRC [62]. IL-6 exerts its effects through two different pathways called classical and trans-signaling pathways (Figure 2A,B). In the classical signaling pathway, IL-6 binds to the membrane-bound receptor IL-6R (mIL-6R), which then transmits the signal through the recruitment and homodimerization of two glycoprotein 130 (gp130) subunits (Figure 2A). Consequently, this leads to phosphorylation and activation of the Janus kinases (JAKs) and STAT3 signaling (Figure 2C). Moreover, the IL-6-activated STAT3 in TME can cause the malignancy of CRC via transcriptional (promoter binding) and post-translational (K116 deacetylation) upregulation of Fructo-Oligosaccharide-like 1 (FOSL1) and FOS-related Antigen 1 (FRA1) [63]. In addition, an increase in STAT3 activation upregulates carcinoembryonic antigen-related cell adhesion molecule 5, which plays a pivotal role in cell adhesion, migration, tumor invasion and metastasis in CRC [62]. Although the gp130 protein is ubiquitously expressed, IL-6R expression is limited to certain types of cells such as hepatocytes and leukocytes. Cells such as endothelial cells, which are not able to secrete mIL-6R, react to IL-6 through the alternative pathway, which is referred as the IL-6 trans-signaling pathway (Figure 2B). In this pathway, a soluble receptor of IL-6 (sIL-6R) is released by alternative splicing or by A Disintegrin and Metalloprotease 17 (ADAM17) protease-mediated receptor shedding from the membrane [47]. The IL-6/sIL-6R complex can bind to gp130 on tumor cells, activate the JAKs and STAT3 signaling and promote tumor cell proliferation and prevent apoptosis [51,64,65,66]. Genetic ablation of IL-6 ameliorates tumor development in an AOM/DSS-elicited colitis-associated cancer model [67]. 

A wide range of therapeutic approaches including anti-IL-6 or anti-IL-6 receptor antibodies, soluble gp130Fc and selective small molecule JAKs inhibitors have been created to block the IL-6/STAT3 pathway for treating human cancers (Figure 2C). Siltuximab (CNTO328, Centocor, Inc., Horsham, PA, USA), an anti-IL-6 antibody, is in phase I/II clinical trials in patients with advanced solid tumors, including CRC [68]. Preclinical experimental studies for the development of novel and potent therapeutics for CRC treatment are in the progress for compounds such as sgp130Fc and CEP-33779 (Cephalon, Inc., Frazer, PA, USA), which are IL-6 trans-signaling and JAK2 inhibitors, respectively [69,70]. 

## 5. Interleukin (IL-1β) 

### 5.1. IL-1β Discovery

IL-1β was first identified in the early 1940s with different names such as Pyrexin, granulocyte pyrogen, circulating endogenous pyrogen, human monocyte pyrogen, lymphocyte activating factor, leukocytic endogenous mediator, mononuclear cell factor and catabolin [71]. In 1979, its nomenclature was interleukin, and in 1984 human IL-1β cDNA was isolated from peripheral blood mononuclear cells [72]. IL-1β is a 31 kDa inactive precursor, and the IL-1β converting enzyme caspase-1 cleaves this precursor between Asp116 and Ala117 to form the active IL-1β precursor with 17 kDa molecular weight [73].

### 5.2. IL-1β Expression and Regulation in Primary Colon Tumor and Hepatic Metastasis Tissue

**Figure 3:**
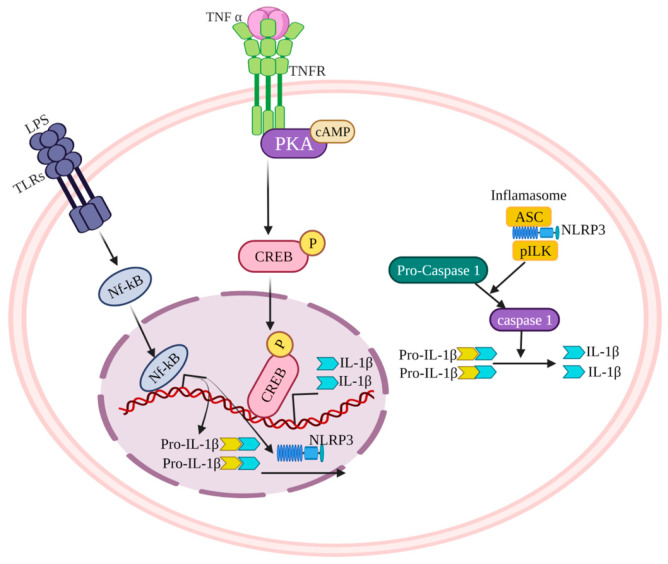
Schematic representation of the IL-1β production via either TNFRs or Toll-like receptors (TLRs) signaling pathways and inflammasome activation. See main text for detailed description.

Macrophages, DCs, monocytes, and neutrophils can all produce IL-1β via either TNFRs or Toll-like receptors (TLRs) signaling pathways [74,75,76] (Figure 3). CD4+ T cells produce TNF-α, which employs TNFRs on mononuclear phagocytes for the transcriptional induction of pro-IL-1β, an inactive 31 kDa protein [77]. Mechanistically, TNF-α induces IL-1β production in the presence of dibutyryl cyclic adenosine monophosphate (cAMP). TNF-alpha/cAMP activates protein kinase A (PKA), which phosphorylates the cAMP-responsive element-binding protein (CREB). Phosphorylated CREB binds to the cAMP-responsive element (CRE) that is situated in the upstream regulatory sequence of IL-1β gene between the 2762 and 2755 base pairs (bp) and induces the transcriptional regulation of IL-1β [78]. Bacterial-derived lipopolysaccharides can bind to TLRs and prime the transcription of IL-1β gene via NF-κB activation. The nucleotide-binding oligomerization domain-containing protein (NOD)-like receptor and pyrin-containing protein 3 (NLRP3) inflammasome activation is critical for IL-1β maturation. Inflammasome activation causes procaspase 1 cleavage to form activate caspase 1, which cleaves pro-IL-1β to produce active 17 kDa IL-1β [79] (Figure 3).

### 5.3. IL-1β Function and Therapeutically Targeting Potential

**Figure 4:**
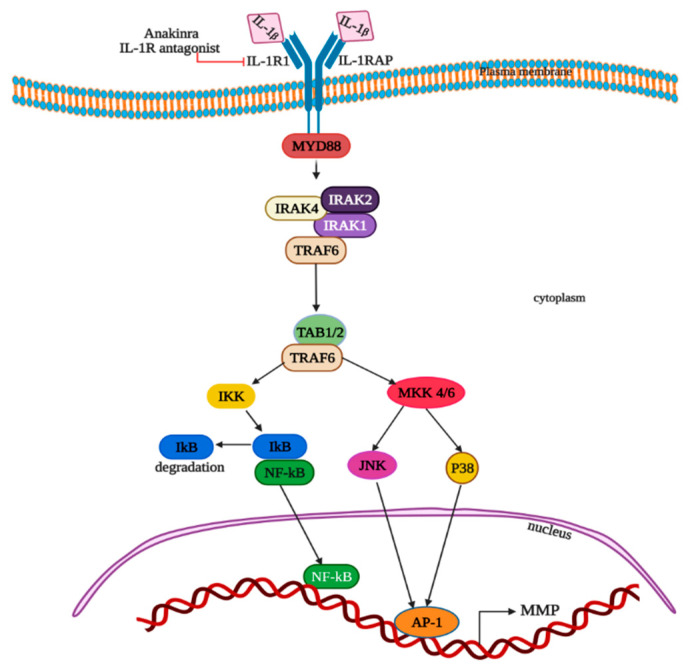
Schematic representation of IL-1β-signaling pathways in CRC. IL-1β stimulates the overexpression of matrix metalloproteinases (MMP) in colon cancer epithelial cells through activation of protein kinases, AP-1 and NF-kB. See main text for detailed description.

Many studies reported that IL-1β plays a pivotal role in tumor growth in CRC and its metastasis [80,81,82,83]. By acting directly on tumor cells, IL-1β can induce tumor cell proliferation [84] and also triggers the stimulation of the MDSCs on tumors to promote the advancement of tumors [85]. Secretion of IL-1β from macrophages, via the stimulation of colon tumor cells, inactivates glycogen synthase kinase 3β and enhances Wnt signaling, which in turn promotes colon cancer cell growth by producing a self-amplifying loop [86,87]. IL-1β stimulates the overexpression of matrix metalloproteinases (MMP) in Caco-2 colon cancer epithelial cells through activation of protein kinases, AP-1 and NF-kB (Figure 4) [88]. Extracellular IL-1 binds to type I transmembrane IL-1 receptor to form a complex with the IL-1 receptor accessory protein (IL1RAP), adaptor MyD88 and Interleukin 1 receptor-associated kinases IRAK-1, IRAK-2 and IRAK-4. IL-1β; binding to receptors leads to the phosphorylation of IRAK-1 on Thr-209 and Thr387 and autophosphorylation on the ProST region (proline-, serine- and threonine-rich), reacting with tumor necrosis factor receptor-associated factor (TRAF6) via TRAF-interacting protein with a forkhead-associated domain (TIFA). This results in the creation of an intermediate complex IRAK-1-IRAK-4-TRAF6. The intermediate complex dissociates after the polyubiquitinylation of TRAF6 and forms active TRAF6. Sequentially, other complexes comprised of IRAK-1, TRAF6, TAK1 and two TAK1-binding proteins TAB1 and TAB2, or alternatively TAK1, TAB1 and TAB3, is generated at the cytoplasmic membrane. Then, the ubiquitinylation and degradation of phosphorylated IRAK-1 occurs in proteasomes. Afterwards, TAB2 phosphorylation, TAK1 autophosphorylation by TAB1 and its activation occurs. Phosphorylated TAK1 and TAB2 translocate the complex to cytosol from the membrane. All these sequential events lead to the phosphorylation of the NF-κB inhibitor (IκB) on -32 and -36 serine residues by mitogen-activated protein kinase kinase 1 (MEKK1), covalent modification by TRAF6-mediated and Ubc13/Uev1A-dependent ubiquitinylation at lysine-63 and the drive to proteasome for degradation. Thus, the generated NF-κB transcription factor enters the nucleus and activates the targeted genes transcription. Alternatively, IL-1β can also trigger the signal via MAP kinases and AP-1, as the TAK1 kinase is capable of triggering the activation of both the MKK4-JNK and MKK6-p38 pathways (Figure 4).

The upregulation of MMP expression was observed in CRC, and this has a correlation with the invasion and metastasis of tumor. IL-1β fosters the epithelial to mesenchymal transition (EMT) process and stem cell proliferation in human primary colon cancer cells and in HCT-116 cells through the zinc finger E-box-binding homeobox 1 (ZEB1) protein, which contributes to the advancement of colon tumors [89]. During the EMT process, epithelial cells acquire a phenotype of mesenchyme tissue and migratory capacity, which plays a pivotal role in cancer metastasis [90,91]. IL-1β activates T-cells and B-cells, which aid in the production of cytokines and antibodies, respectively [92]. IL-1β also induces the synthesis of prostaglandins (PG), proliferation of fibroblasts and production of collagen. IL-1β aids in the synthesis of interferon-γ from T-helper cells by synergizing with IL-12 and also promotes the differentiation of Th17 cells into Il-17+IFN-γ + Th cells [93,94,95]. IL-1β can also promote colon tumor growth and metastasis through activating inflammasome and inducing angiogenesis at the sites of primary tumors and metastasis [96].

Tumor growth and metastasis inhibition can be achieved by the blockage of the IL-1 receptor (IL-1R) with IL-1R antagonist. Genetic ablation of IL-1R in intestinal epithelium, and T-cells alleviated tumor formation and progression in the mouse models of CRC, which provides evidence that IL-1R signaling aids in the advancement of CRC [97]. Inhibitors at different levels of the IL-1β synthesis and signaling pathways help in CRC therapy [98]. The human IL-1 receptor antagonist Anakinra inhibits both IL-1β and IL-1α and reduces metastatic CRC in an IL-17- and IL-22-dependent manner (Figure 4) [99].

## 6. Lipid Molecule PGE2

### 6.1. PGE2 Discovery

The prostaglandins, otherwise called eicosanoids, are active functional lipid molecules which possess a wide range of hormonelike effects in living beings. PGE2 is one among these functional eicosanoids and was first identified in sheep’s seminal vesicle by the Swedish biochemist and Noble laureate Sune Bergstrom along with his co-researchers in 1962 [100]. Later, PGE2 was synthesized as racemic mixture in 1969 and as natural isomer in 1970 [100,101]. As PGE2 exerts action at the site of synthesis, it is known as autacoid. 

### 6.2. PGE2 Expression and Regulation in Primary Colon Tumor and Hepatic Metastasis

**Figure 5:**
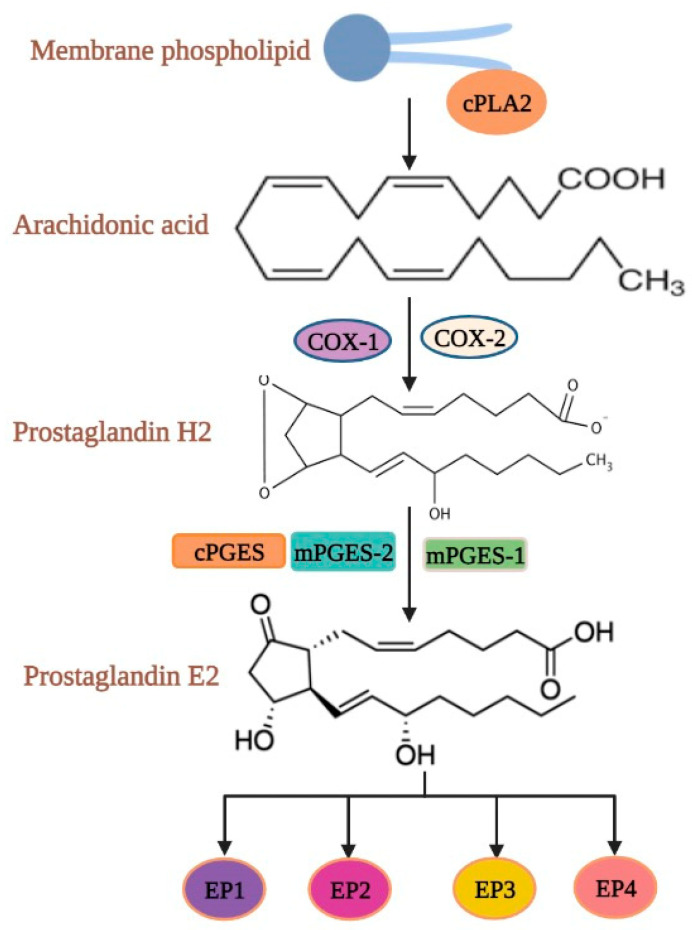
Synthesis of PGE2 by the oxidative cyclisation of Arachidonic acid via Cyclooxygenases. COX-1 and COX-2 convert free arachidonic acid to PGH_2_. PGH2 is further metabolized to PGE2 by PGES.

Cytosolic phospholipase A2α (cPLA2α) involves in the catabolism of plasma membrane phospholipids to produce arachidonic acid (AA) [102,103,104,105] (Figure 5). The oxidative cyclisation of AA by cyclo-oxygenase (COX) enzymes leads to the synthesis of PGH2 [106]. PGH2 is then transformed into PGE2 by microsomal prostaglandin E synthase-1 (mPGES-1), mPGES-2 and cytosolic PGES (cPGES) [107]. PGE2 is expressed in almost all nucleated cells. PGE2 synthesis was enhanced by the upregulation of COX-2 enzymes in HT29 and HCT116 CRC cell lines, where the increased PGE2 stimulates VEGF production and enhances the tumor cell survival rate during hypoxia [108]. In CRC patients, the overexpression of COX-2 and PGE2, and PGE2 binding with G protein coupled PGE2 receptors 1–4 (E-type prostanoid receptors; EP1-4) on endothelial cells in tumor blood vessels, has been observed, directly affecting tumor angiogenesis by increasing cell survival [109]. We showed that HIF-2α can induce the expression of COX2 and mPGES-1 to promote PGE2 production in CRC [110]. A recent study reported that the decreased expression of EP4 receptor in HCA-7 cells via interleukin-4 triggers the downregulation of COX2 and PGE2 expression [111]. 

### 6.3. PGE2 Function and Therapeutic Targeting Potential

**Figure 6:**
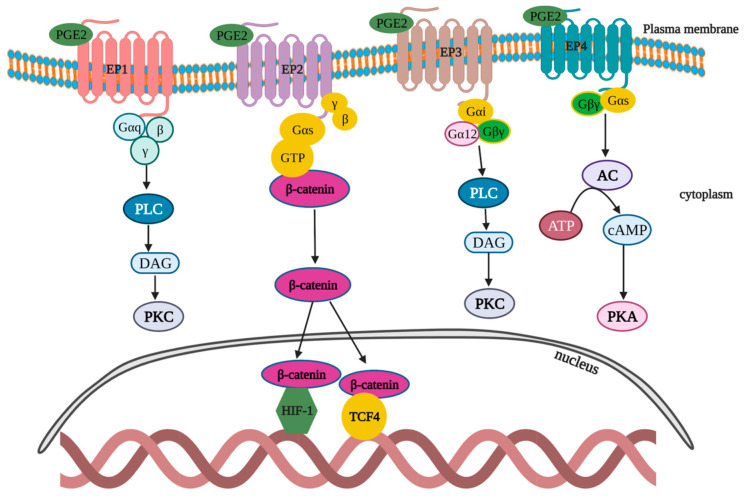
Schematic representation of PGE2-signaling pathways in cells. PGE2 acts in both an autocrine and paracrine manner by binding with EP1-4, activating various protein kinase pathways.

PGE2 signaling is one among the critical pathways that govern tumor progression and immune dysfunction [112]. PGE2 acts in both an autocrine and paracrine manner by binding with EP1-4 (Figure 6). EP1: After binding with the EP1 receptor, PGE2 stimulates and mobilizes Gαq-Gβγ proteins, which triggers phospholipase C (PLC) to subsequently generate and activate diacylglycerol (DAG), and thereby upregulates protein kinase C (PKC). PKC activation exerts a negative feedback effect to desensitize the EP1 activation. In addition to the aforementioned pathway, EP1 can trigger p38 mitogen-activated protein kinases (MAPK), ERK and CREB pathways [113]. EP2: After binding with EP2 receptor, PGE2 mobilizes the Gαs–Gβγ complex to promote the nucleus translocation of β-catenin from cytoplasm and the activation of HIF1 and T cell factor-4 (TCF4)-signaling pathways [114,115]. EP3: Upon binding to EP3 receptors, PGE2 induces the activation of a wide range of G protein complexes (Gαi–Gβγ and Gα12–Gβγ), which in turn activates PLC to subsequently generate DAG and activate PKC [116]. EP4: PGE2 binds to the EP4 receptor and stimulates the induction of the Gαs–Gβγ complex that leads to the sequential steps of adenyl cyclase (AC) activation, which catalyze the formation of cAMP from ATP, thus causing PKA activation [117]. 

It was extensively recorded that PGE2 exhibits a variety of biochemical effects related to inflammation and induces apoptosis, angiogenesis, immuno-monitoring and cell proliferation in tumor cells. PGE2 derived from COX-2 promotes the growth of CRC via the activation of epidermal growth factor receptor (EGFR) signaling [10,114]. In myeloid DCs differentiated from bone marrow, PGE2 is important in modulating the secretion of cytokines such as TNF-α, IL-6 and IL-23 by reorienting the cell’s differentiation and maturation [118]. Multiple studies reported the inhibition of DCs’ function by PGE2 [118,119,120]. One study reported that PGE2 inhibited murine bone-marrow-derived DCs’ function by inhibiting its antigen-presenting potential [121]. PGE2 re-orients the differentiation of DCs to immuno-suppressor cells, including MDSCs, which promotes tumor cell growth. Exogenous PGE2 treatment promotes CRC progression and hepatic metastasis in *Apc*^Min/+^ mice [122]. Elevated levels of plasma PGE2 were reported in CRC patients, which indicates the role of PGE2 as CRC promoter component [123]. It was thoroughly studied that PGE2/EP signaling is the most essential step in CRC angiogenesis, and this signaling pathway also aids in PGE2 secretion, which plays a major role in CRC progression. 

O’Callaghan et al. reported that PGE2 triggers the upregulation of FasL expression via EP1 receptors, which promotes CRC progression [124]. Another recent in vivo study reported that PGE2 can also activate colonic cancer stem cell (CSC) proliferation and liver metastasis via multiple signaling pathways, such as NF-κB, phosphoinositide 3-kinase (PI3K)-AKT and MAPK, by binding to its EP2 cell surface receptors [125]. Löffler et al. reported that PGE2-mediated long-term EP3 receptor activation triggers CRC cell proliferation [126]. Another study reported that PGE2 binding with EP4 receptors stimulates the secretion of TNF-α by activating TLR and disturbs the Th1 to Th2 cytokine shift in T cells [112].

Nonsteroidal anti-inflammatory drugs (NSAIDs), including indomethacin, aspirin, sulindac and piroxicam, are traditional COX1 and COX2 inhibitors used in CRC prevention [127]. A reduction in PGE2 was reported in human primary CRC LS-174T cells treated with EP4 antagonist ONOAE-208 via the activation of NF-kB through EP4-PI3K and EP4-MAPK pathways [122]. An HSP-90 inhibitor (17-demethoxy-17- allylamino geldanamycin, 17-AAG) was also reported for its inhibitory effects on the levels of PGE2 in CRC HT-29 cells through the inhibition of COX2 [128]. A research report proposed that SLCO2A1, a prostaglandin transporter, plays a significant role in maintaining the concentration and release of PGE2 and also stated that SLCO2A1 pharmacological intervention may possess robust scope for developing CRC therapeutics [129].

## 7. Chemokines

Chemokines are tiny protein molecules, called chemo-attractant cytokines, that play a critical role in the migration of cells from the blood to the tissues and vice versa. Chemokines also trigger the movement in the consequence of a chemical gradient by a process called chemotaxis. Moreover, chemokines regulate and mediate the development of lymphoid organs, differentiation of T-cells and metastasis of tumor cells. The first chemotactic chemokine was discovered in 1977 [130]. Chemokines exert their effects via binding to chemokine receptors which belong to the G-protein-coupled receptor family. They are classified or grouped based on two different criterions: (1) based on the amino acid composition (especially on the first two cysteine residues of a conserved tetra-cysteine motif), and they are grouped as CC and CXC chemokines; (2) based on function, and they are grouped as inflammatory or homeostatic chemokines. Furthermore, the CXC chemokines are categorized as angiogenic or angiostatic chemokines based on the possession of glutamic acid–leucine–arginine (ELR) motif. The CXC chemokines which are ELR motif positive tend to be angiogenic, and when ELR motif negative, tend to be angiostatic chemokines. 

## 8. CXCL1

### 8.1. CXCL1 Discovery 

CXCL1 belongs to the CXC chemokine family that binds to CXC receptor 2 (CXCR2), a G-protein-coupled receptor to exert its effects. Previously, CXCL1 was named as growth-regulated oncogene alpha (Gro-α), GRO1, melanoma growth-stimulating activity and neutrophil-activating protein 3 [131]. During the 1980s CXCL1 was initially identified and isolated from culture supernatants of human melanoma Hs0294 T cells, and the CXCL1 gene was located on chromosome 4 (region q13→q21). Human CXCL1 inactive precursor protein comprises 107 amino acids and weights ~11 kDa [132]. The active or mature CXCL1 has 3 isoforms with a maximum length of 73 amino acids. CXCL1 exists in monomer and dimer forms. The monomer comprises three antiparallel β strands and a C-terminal α helix. The globular dimer formation involves the first β strand and the C-terminal α helix in monomer [133].

### 8.2. CXCL1 Expression and Regulation in Primary Colon Tumor and Hepatic Metastasis

CXCL1 is primarily produced by TNF-stimulated endothelial cells and pericytes and aids luminal neutrophil crawling [134]. In addition, a diverse array of immune cells such as Th17 cells, macrophages, neutrophils and epithelial cells produce CXCL1 [135,136,137]. A range of reports were published regarding the overexpression of CXCL1 in colorectal adenocarcinomas [9,138,139,140,141,142,143]. The hypersecretion of CXCL1 in K-ras mutant human CRC epithelial cells and murine fibroblasts was also reported [9]. 

In vitro study in human vascular endothelial cells reveals that TNF-α activates the JNK/AP-1 signaling pathway, which drives the transcription of CXCL1 [144]. A recent report suggested that a drop in the levels of SMAD4 in human CRC cells resulted in the overexpression of CXCL1 mRNA [145]. We also proved that the CXCL1 gene is a novel HIF-2α target gene [146]. The epithelial HIF-2α-induced CXCL1 transcription triggers a robust accumulation of neutrophils and promotes colon tumor progression through CXCR2 binding.

### 8.3. CXCL1 Function and Therapeutic Targeting Potential

**Figure 7:**
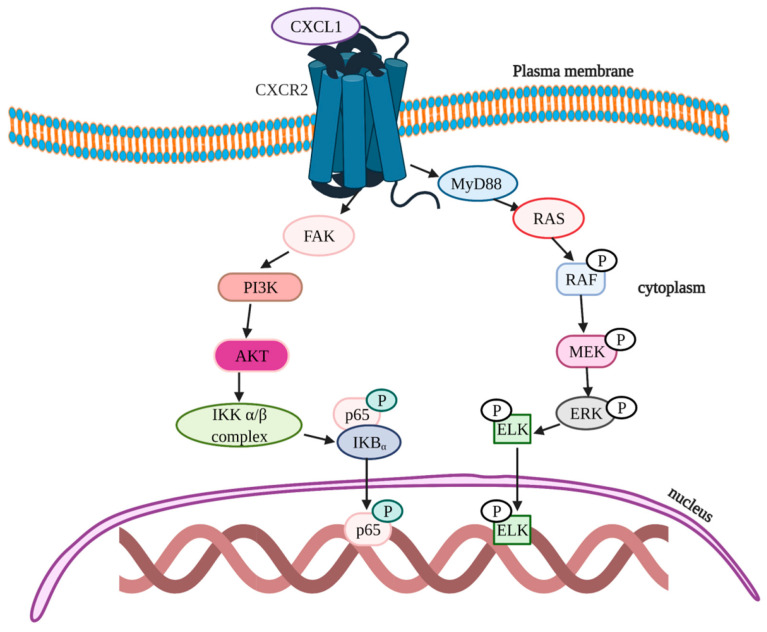
Schematic illustration of signaling pathways of CXCL1 in CRC. CXCL1 activates FAK-PI3K-Akt signaling, which in turn activates the IKKα/β complex and triggers p65 nuclear translocation via the activation of IκBα for tumor cell proliferation; CXCL1 also triggers the activation of MyD88-RAS-RAF signaling, which in turn aids in the activation of the MEK-ERK-ELK signaling pathway.

A wide range of evidence is available to show that the inflammatory chemokines act as tumor-promoting and metastatic factors by enhancing angiogenesis and suppressing immune-mediated tumor eradication [147,148,149]. CXCL1 plays a major role in the establishment of premetastatic niches in CRC hepatic metastasis [150,151]. The elevated levels of CXCL1 are positively associated with poor survival rate in CRC [141]. One study reported the CXCL1 contribution in the formation of hepatic premetastatic niches. CXCL1 can directly recruit circulatory CXCR2-expressing neutrophils and MDSCs from the circulation into inflammatory sites and tumor tissues, promoting liver metastasis [152]. Another study reported that overexpression of CXCL1 stimulates CXCR2^+^ endothelial cell migration and increases the formation of tumor microvessels in CRC patients [153]. Another study demonstrated that excess circulation of CXCL1 leads to a reduction in an extracellular matrix tumor-suppressor protein fibulin-1 through NF-κB/Histone Deacetylase 1 (HDAC1) epigenetic regulation, which facilitates the invasion and metastasis of CRC cells [154,155]. Fibulin1 plays a crucial role in tumor suppression by binding to the fibronectin and through modulating focal adhesion kinase (FAK) and ERK1/2 signaling, which results in the inhibition of cell adhesion and spreading [156] (Figure 7). CXCL1 stimulates FAK, PI3K and Akt which in turn activates the IKKα/β complex to trigger p65 via the activation of IkBα for tumor cell proliferation. CXCL1 also triggers the activation of MyD88-RAS-rapidly accelerated fibrosarcoma (RAF) kinase signaling, which in turn aids in the activation of mitogen-activated extracellular signal-regulated kinase (MEK)-ERK-Ets-like protein (ELK) pathways that leads to CRC [157] (Figure 7).

The receptor CXCR2 is the prime target to develop potent therapeutics to reduce the action of CXCL1 in CRC hepatic metastasis. The receptor tyrosine kinase inhibitor TSU68 reduces the translocation of the bone-marrow-derived endothelial progenitor cells into the premetastatic liver and suppresses endothelial cell production of CXCL1 [150]. As a result, CXCR2^+^ neutrophils recruitment, angiogenesis and hepatic metastasis are reduced in mice [150]. Two small molecule CXCR2 antagonists, SCH-527123 and SCH-479833, were reported to be robust antimetastatic therapeutics in human CRC liver metastasis, which acts at the metastatic site by declining the tumor vascularization and enhancing the malignant cell apoptosis [158]. 

## 9. CXCL2

### 9.1. CXCL2 Discovery

The chemokine ligand 2 (CXCL2) belongs to the CXC motif chemokine family, and its synonyms are macrophage inflammatory protein 2-alpha (MIP2-alpha), Melanoma Growth-Stimulating Activity Beta (MGSB), Growth-Related oncogene-2 (Gro-2/ Gro-beta), Small inducible Cytokine Subfamily B member 2 (SCYB2) and Cytokine-Induced Neutrophil Chemo-attractant 2 Alpha (CINC2-α) [159,160]. In 1988, CXCL2 was isolated from human melanoma cell line culture supernatants [156]. The gene is localized on chromosome 4 q21 [161,162]. CXCL2 shares 90% amino acid sequence similarity and 64% sequence identity with CXCL1 [163]. Similar to CXCL1, the inactive form of CXCL2 comprises 107 amino acids and weighs ~11.389 kDa. By the membrane type 6-matrix metalloproteinase enzyme proteolytic cleavage of 34 amino acid residues of inactive precursor protein produces the active form of CXCL2 (5-73 aa) [164,165]. Similar to CXCL1, CXCL2 exerts its activity via high-affinity binding to CXCR2 as well.

### 9.2. CXCL2 Expression and Regulation in Primary Colon Tumor and Hepatic Metastasis

**Figure 8:**
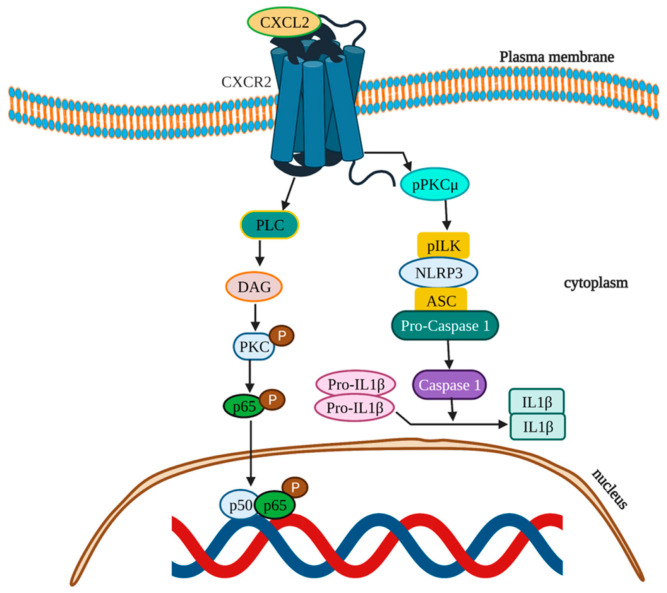
Schematic illustration of signaling pathways of CXCL2 in CRC. CXCL2 activates the NF-kB pathway via PLC-DAG-PKC signaling to promote angiogenesis and tumor growth; CXCL2 also activates pPKCμ to promote NLRP3 inflammasome activation and IL-1β secretion during tumorigenesis.

CXCL2 is primarily produced by neutrophils, and this chemokine is required for unidirectional paracellular neutrophil transendothelial cell migration in vivo [134]. Significant upregulation in the CXCL2 expression was noticed in the CRC tissues and in CRC liver metastasis [166]. CXCL2 expression can be induced by a variety of inflammatory mediators such as TNFα, IL-1β and lipopolysaccharide in endothelial cells, fibroblasts, melanocytes, monocytes and megakaryocytes [161]. CRC treatment with chemotherapeutic agents induces the synthesis of cytokine TNF-α, which in turn triggers the paracrine release of CXCL2 and results in tumor promotion and chemo-resistance [167]. In HT-29 and HCT116 CRC cells, tumor growth and angiogenesis was promoted by guanine the nucleotide-binding protein alpha-13 (GNA13) via upregulation of CXCL2 through the activation of the PLC-DAG-PKC-NF-kB signaling pathway (Figure 8) [168]. It was also reported that CXCL2 secreted by colorectal cancer stem cells (CRCSCs) from murine CRC cell line CT26 attracts the neutrophils and promotes the tumorigenesis through NLRP3 inflammasome dependent IL-1β secretion (Figure 8) [169]. 

### 9.3. CXCL2 Function and Therapeutic Targeting Potential

CXCL2 binds to CXCR2, which in turn couples with Gαi to stimulate neutrophil chemotaxis and endothelial cell migration for tumor transformation and growth in CRC cells. In a study on LoVo colon cancer cells, it was elucidated that the CXCL2–CXCR2 axis induces cancer stem cell properties and metastasis by the activation of Gαi and Gαq/11 [170]. MDSCs are attracted by the expression of CXCL2 in the TME and trigger the enhancement of cancer cell survival.

CXCL2 executes a pivotal role in numerous biochemical processes such as inflammation, immune response, angiogenesis and wound healing. The overexpression of CXCL2 promotes CRC tumor progression and liver metastasis [162,169,170,171]. A recent report suggests that miR-532-5p, a tumor-suppressor miRNA, negatively regulates CXCL2 in liver cancer cells. They have also reported that CXCL2 downregulation induces apoptosis and inhibits proliferation of tumors, which in turn downregulates the expression of a wide range of metastasis-promoting factors, including Secreted Protein and Rich in Cysteine (SPARC), Epidermal growth factor-containing fibulin-like extracellular matrix protein (EFEMP) and COX2 in CRC [172]. In other reports, CXCL2 dose-dependently increases the expression of VEGF within the angiogenic front of tumor margins, thereby promoting tumor angiogenesis, tumor growth and hepatic metastasis in the CXCR2-expressing CT26 CRC cell-induced CRC mice [160,173]. Similar to CXCL1, CXCR2 inhibition decreases the CXCL2-induced neovascularization and tumor progression [158]. The CXCR2 antagonist AZD5069 was reported to inhibit the overexpression of CXCL2, thereby inhibiting the GNA13-induced vascular endothelial growth [169]. 

## 10. Concluding Remarks

Immunity and cancer-promoting proinflammatory microenvironments are two key cancer traits that contribute to cancer formation [174]. Cytokines and chemokines in the TME trigger the activation of transcription factors for tumor advancement and metastasis [174,175]. In this review, we discussed that three cytokines, a lipid molecule PGE2 and two chemokines are overexpressed in CRC cells and liver metastasis. Findings from this review disclose that a wide range of cytokine and chemokine inhibitors are employed in preclinical studies for targeted therapy to prevent CRC invasion and liver metastasis. These proinflammatory cytokines and chemokines that increase various transcription factors have been recognized as potential cancer therapy targets. Systemic clinical trials are in dire need to confirm their potency in CRC patients and to evaluate their beneficial effects as single or combinational therapy.

## Abbreviations

AA: arachidonic acid; AP-1, activator protein-1; ASC, apoptosis-associated specklike protein containing caspase recruitment domain; BSF-2, B-cell stimulatory factor 2; CRC, colorectal cancer; cAMP, cyclic adenosine monophosphate; CXCL1/2,CXC chemokine ligand 1/2; COX, cyclo-oxygenase; DC, dendritic cells; EMT, epithelial to mesenchymal transition; EP1-4, E prostanoid receptor 1-4; ERK, extracellular signal-regulated kinase; FAK, focal adhesion kinase; FOSL1, fructo-oligosaccharide-like protein 1; FRA1, FOS-related Antigen 1; GNA13, guanine nucleotide-binding protein alpha-13; HIF-2α, hypoxia-inducible factor-2 alpha; IL-1β, interleukin-1β; IL-6, interleukin-6; IKKβ, inhibitor of nuclear factor kappa B Kinase beta; JNK, c-Jun N-terminal kinases; MAPK, mitogen-activated protein kinases; MDSC, myeloid-derived suppressor cells; MMP, matrix metalloproteinases; NF-kB, nuclear factor kappa-light-chain-enhancer of activated B cells; NLRP3, nucleotide-binding oligomerization domain-containing protein or NOD-like receptor and pyrin-containing protein 3; PDGF, platelet-derived growth factor; PGE2, prostaglandin E2; PI3K, phosphoinositide 3-kinase; pILK, phospho-integrin-linked kinase; PKC, protein kinase C; STAT3, signal transducer and activator of transcription-3; TAMs, tumor-associated macrophages; TME, tumor microenvironment; TNF-α, tumor necrosis factor alpha; TNFR1, tumor necrosis factor receptor1, VEGF, vascular endothelial growth factor; Zeb1, zinc finger E-box-binding homeobox 1.
